# Urinary Metabolites of Organophosphate Pesticides among Pregnant Women Participating in the Japan Environment and Children’s Study (JECS)

**DOI:** 10.3390/ijerph18115929

**Published:** 2021-05-31

**Authors:** Yukiko Nishihama, Shoji F. Nakayama, Tomohiko Isobe, Chau-Ren Jung, Miyuki Iwai-Shimada, Yayoi Kobayashi, Takehiro Michikawa, Makiko Sekiyama, Yu Taniguchi, Shin Yamazaki, on behalf of the Japan Environment and Children’s Study Group

**Affiliations:** 1 Japan Environment and Children’s Study Programme Office, Health and Environmental Risk Division, National Institute for Environmental Studies, Tsukuba 305-0053, Ibaraki, Japan; nishihama.yukiko@nies.go.jp (Y.N.); isobe.tomohiko@nies.go.jp (T.I.); jung.chau-ren@nies.go.jp (C.-R.J.); iwai.miyuki@nies.go.jp (M.I.-S.); kobayashi.yayoi@nies.go.jp (Y.K.); takehiro.michikawa@med.toho-u.ac.jp (T.M.); sekiyama.makiko@nies.go.jp (M.S.); taniguchi.yu@nies.go.jp (Y.T.); yamazaki.shin@nies.go.jp (S.Y.); 2 Department of Public Health, College of Public Health, China Medical University, Taichung City 406040, Taiwan; 3 Department of Environmental and Occupational Health, School of Medicine, Toho University, Tokyo 143-8540, Japan

**Keywords:** organophosphate pesticides, dialkylphosphates, urine, biomarker, pregnant women, birth cohort, cumulative risk assessment, relative potency factors

## Abstract

Organophosphate pesticides (OPPs) exhibit neurodevelopmental toxicity. To evaluate the effect of prenatal exposure to OPPs in the Japan Environment and Children’s Study, a nationally representative birth cohort study, 4575 maternal urine samples were analysed for six OPP metabolites, i.e., dialkylphosphates (DAPs). This study aimed to investigate predictors of urinary DAPs using machine learning approaches and to assess the cumulative risk based on relative potency factors among Japanese pregnant women. The median creatinine-normalised urinary concentrations (interquartile ranges) of dimethylphosphate, dimethylthiophosphate and diethylphosphate, which had a detection rate of 50% or higher, were 3.53 (1.91–6.78), 4.09 (1.66–10.8) and 3.28 (1.88–5.98) µg/g-creatinine, respectively. Possible predictors of urinary DAP concentrations were the month of urine sampling, consumption of apple and maternal body mass index. When fenitrothion was used as an index chemical for cumulative risk assessment, 0.36% of participants exceeded the lower 95% confidence limit of the benchmark dose_10_.

## 1. Introduction

Organophosphate pesticides (OPPs) are irreversible acetylcholinesterase (AChE) inhibitors and thus used worldwide as insecticides; however, they also exhibit brain developmental toxicity and neurotoxicity [1,2]. Recently, increasing numbers of epidemiological studies have demonstrated the impacts of OPPs on neurodevelopment in children [3,4,5] and via prenatal exposure [6]. Some studies reported that human exposure to OPPs is associated with the season; intake of certain food items, such as vegetables (tomato and sweet pepper), fruits (apple, banana, citrus, kiwi and apricot), beans, bread and drinking water; and the use of pesticides in workplaces [7,8,9]. Similar results were obtained from large-scale birth cohort studies [10,11].

In Japan, the OPPs most frequently used in agricultural and residential areas are fenitrothion, acephate and diazinon [12]. Their maximum residue limits are higher in Japan than in European Union countries [13]. Use of OPPs has gradually decreased in Japan [14].

To evaluate OPP exposure, urinary concentrations of common OPP metabolites, i.e., dialkylphosphates (DAPs) including dimethylphosphate (DMP), diethylphosphate (DEP), dimethylthiophosphate (DMTP), diethylthiophosphate (DETP), dimethylditiophosphate (DMDTP) and diethyldithiophosphate (DEDTP), are usually used as biomarkers in cohort studies [15]. OPPs with mono-thio or di-thio moieties have been reported to be metabolised by three kinds of DAPs, i.e., dialkyl, dialkylthio and dialkyidithio phosphates [16]. Many OPPs share the mono-thio or di-thio moieties, thus DAPs do not represent any specific OPPs. Biological half-lives of OPPs and DAPs have been reported to be 12–36 and 2–15.5 h, respectively [17,18,19]; thus, DAPs should be considered as biomarkers of short-term exposure. There are no nationally representative biomonitoring data for OPPs in Japan. A few studies conducted in the last five years showed that metabolites of OPPs were detected in Japanese women and children [14,20], including pregnant women [21].

The U.S. Environmental Protection Agency (U.S. EPA) developed a method for risk assessment of chemical mixtures with similar properties (structure) and calculated relative potency factors (RFPs) for cumulative risk assessment of OPPs using oral benchmark dose (BMD) values based on a reduction in brain cholinesterase activity [22,23,24]. The BMD method applies a mathematical model to the relationship between the incidence of toxicity (numerical changes such as weight loss or the frequency of onset of toxicity) and the exposure dose, and, in the most statistically fit model, calculates the lower confidence limits of the amount of exposure for a dose of the benchmark response (BMR), which detects the significant effects analysed in the experimental systems as a BMD lower confidence limit (BMDL). Commonly, 10% onset of toxicity is used for general toxicity as the BMR, i.e., BMD_10_. RFP indicates the relative potency of each toxicant, such as BMD_10_. To our knowledge, one study has assessed the risk of OPP exposure in pregnant women using this method [25], while some studies have assessed the risk of OPP exposure using other methods [26,27,28,29,30].

No previous study has investigated the determinants of urinary OPP metabolites and the cumulative risk of OPP exposure in Japanese pregnant women. This study aimed to investigate possible predictors of urinary OPP metabolite concentrations among Japanese pregnant women and to conduct a cumulative risk assessment for exposure to a mixture of OPPs [25].

## 2. Materials and Methods

### 2.1. Study Participants and Sample Collection

This study was conducted in the framework of the Japan Environment and Children’s Study (JECS), an ongoing nationally representative birth cohort study whose protocol and profile were published in detail previously [31,32]. JECS recorded 103,099 pregnancies from January 2011 to March 2014 in 15 study areas across Japan. The JECS protocol was reviewed and approved by the Institutional Review Board on Epidemiological Studies of the Ministry of the Environment on 6th April 2010 (IRB number: 100406001) and by the Ethics Committees of all participating institutions. Written informed consent was obtained from all participants. This study used the JECS dataset which includes study data from pregnancy to 4 years (*n* = 104,059; jecs-qa-20210401). OPP metabolites were measured in 4575 maternal urine samples during pregnancy. The subjects were the participants of the Sub-Cohort Study [33] who had urine samples when they were registered. Seventy-eight women were excluded from the current study because of their withdrawal from the study and 38 women were excluded due to missing serum creatinine data. A total of 4456 women were included in this study (Supplementary Figure S1). Detailed information about urine collection and storage before analysis was presented in the previous publication [34].

### 2.2. Chemicals and Reagents

All reagents were of high-quality grade unless specified otherwise. Water was brought to a total organic carbon concentration of ≤15 ppb using a Milli-Q Integral 5 and MT5 system (Merck Millipore, Burlington, MA, USA). Acetonitrile (99.8% purity), ammonium acetate and formic acid were purchased from FUJIFILM Wako Pure Chemical Corporation (Osaka, Japan). A standard solution of O,O-dimethylphosphoric acid potassium salt (DMP), O,O-diethylphosphoric acid potassium salt (DEP), O,O-dimethylphosphorothioate potassium salt (DMTP), O,O-diethylphosphorothioate potassium salt (DETP), O,O-dimethylphosphorodithioate potassium salt (DMDTP) and O,O-diethylphosphorodithioate potassium salt (DEDTP), as well as an internal standard (IS) solution containing DMP-d_6_, DEP-d_10_, DMTP-d_6_, DETP-d_10_, DMDTP-d_6_ and DEDTP-d_10_ (98% purity), were purchased from Cambridge Isotope Laboratories, Inc. (Tewksbury, MA, USA).

### 2.3. Sample Preparation

Twenty-five microlitres of a centrifuged urine sample and 25 µL of water were added to an ISOLUTE FILTER+ Plate 96-well plate containing a 25 µm depth filter and 0.2 µm wettable membrane filter (Biotage, Uppsala, Sweden), together with 10 µL of 125 ng/mL IS solution, 5 µL of water and 805 µL of acetonitrile. After incubation for 1 h in a refrigerator at 4 °C, the plate was centrifuged (4 °C, 2200× *g* for 2 min) and the supernatant was injected into a high-performance liquid chromatography-tandem mass spectrometer (LC-MS/MS) (Supplementary Figure S2).

### 2.4. Instrument Analysis and Calculations

The LC (Nexera X2 system; Shimadzu, Corporation, Kyoto, Japan) and MS/MS (Triple Quad 6500; AB Sciex Pte. Ltd., Framingham, MA, USA) systems were operated using electrospray ionization negative mode with multiple reaction monitoring. For the measurement of DMP and DEP, the analytical column was the Luna HILIC 200 Å, 2.0 mm I.D. × 100 mm, 5 µm column (Phenomenex, Torrance, CA, USA), the column flow rate was 0.4 mL/min, while for measurement of other metabolites, a clean-up column was used, i.e., Scherzo SM-C18, 2.0 mm I.D. × 100 mm, 3 µm column (Imtakt Corp., Kyoto, Japan) followed by an analytical column, Luna HILIC 200 Å, 2.0 mm I.D. × 100 mm, 5 µm column (Phenomenex). The column flow rate was 0.3 mL/min. A column-switching technique was used for DMTP, DETP, DMDTP and DEDTP measurement (Supplementary Figure S3). The column temperature was kept at 40 °C for both measurements. The typical routine operating conditions and data acquisition settings are shown in Supplementary Tables S1–S3. The following precursor ion (*m/z*)/product ion (*m/z*) combinations were used for the detection of DMP, DMTP, DMDTP, DEP, DETP and DEDTP: 125.0/63.0, 141.0/96.0, 157.0/142.0, 153.0/125.0, 169.0, 95.0 and 185.0/111.0, respectively (Table S2). Parameters of MS/MS were optimised using reference standard solutions, e.g., −4500 V for ionspray voltage, 500 °C for heating gas temperature and 11 for collision gas pressure (Table S3). The calibration range is documented in Supplementary Table S4. All samples outside the calibration range were re-analysed after further dilution.

A commercially available urine sample collected from a female donor (BioIVT, Westbury, NY, USA) was received as a reference standard solution to make a quality control (QC) sample of 20 ng/mL DAP concentrations. The QC sample was analysed in five replicates in each analytical sequence. The lowest concentration minimum reporting level (LCMRL) was calculated according to the U.S. EPA’s instructions [35]. The minimum reporting level (MRL) was set at the lowest concentration of the calibration curve point that observed ≤ ±5% precision (0.997 ng/mL) or the LCMRL, whichever was higher.

### 2.5. QC

Repeatability and intermediate precision were determined based on ISO 5725:1994 and 27148:2010, with standard solution (10 ng/mL including 50 ng/mL IS solution) measurements (*n* = 235–375 for each DAP). QC for day-to-day analysis was determined using a Shewhart control chart ($\bar{X}$-R control chart) according to ISO 7870. A urine sample of the German External Quality Assessment Scheme (G-EQUAS) 61 was measured using this method for external validation. The urinary creatinine concentration was analysed using an enzymatic assay at a contract laboratory. Duplicate measurements were performed in every 50 samples.

### 2.6. Data Collection

Participants were asked to complete two questionnaires, one during the first trimester (12–16 weeks of gestation; M-T1) and the other during the second or third trimester (22–28 weeks of gestation; M-T2). Smoking status was scored on the M-T1 questionnaire as “Never,” “Previously did, but quit before realising current pregnancy,” “Previously did, but quit after realising current pregnancy” or “Currently smoking.” Annual household income was reported as <2 million Japanese yen (~18,181 USD; 1 USD ≃ 110 yen), 2 to <4 million yen (~36,363 USD), 4 to <6 million yen (~54,545 USD), 6 to <8 million yen (~72,727 USD), 8 to <10 million yen (~90,909 USD), 10 to <12 million yen (~109,090 USD), 12 to <15 million yen (~136,363 USD), 15 to <20 million yen (~181,818 USD) and ≥20 million yen. Education was defined as ≤ 12 years or ≥ 13 years as reported on the M-T2 questionnaire. Consumption of foods was estimated using a food frequency questionnaire on the M-T1 questionnaire [36]. The frequency of insecticide, herbicide and pesticide use was scored on the M-T1 and M-T2 questionnaires [37] as no use, 1–3 times a month, 1–6 times a week and every day. The M-T2 questionnaire asked for additional information, i.e., use of a moth repellent for clothes in the closet (never, yes, sometimes and yes, continuously); smoke insecticide indoors (no/yes); and a mosquito coil or electric mosquito repellent mat, a liquid insecticide for maggot and mosquito larva and an herbicide or a gardening pesticide in a garden, balcony or farm (no use, less than once a month, 1–3 times a month, once a week, a few times a week and every day). Maternal age at urine sampling, body mass index (BMI) and gestational age were determined from individual medical record transcripts, maternal consent form and prenatal care records. Median (interquartile range) of maternal age and BMI were 32 (28–35) years old and 20.7 (19.1–22.5) kg/m^2^ (Supplementary Table S5). eGFR was calculated using the following Formula (1) [38]:

(1) $$194\times {\mathrm{serum}\;\cot\mathrm{inine}\;\mathrm{concentration}}^{-1.094}\times {\mathrm{maternal}\;\mathrm{age}}^{-0.287}\times 0.739$$

### 2.7. Data Analysis

Urinary DAP concentrations normalised relative to creatinine concentrations were log10-transformed for statistical analysis. Descriptive statistics of DAP concentrations were calculated using the Kaplan-Meier method in the NADA package (version 1.6-1.1) without substituting data below the MRL [39]. DEDTP was only detected in two participants and was thus excluded from further analysis. DAP concentrations below the MRL were imputed using the quantile regression approach for the left-censored missing (QRILC) method within the Gibbs sampler based on the left-censored missing value imputation approach [40]. Missing data for the variables collected by the questionnaires were imputed using the multiple imputation by chained equations (MICE) method with 15 imputations and 10 iterations. The maximum proportion of incomplete cases was 12%; thus the number of imputations was set to 15 according to the previously published instruction [41]. Majority voting was performed to combine the 15 imputed datasets.

Four machine learning approaches, namely, multivariate linear regression analysis, random forest regression (RFR), gradient boosting machine (GBM) and neural network analysis, in the h2o package (version 3.32.0.1) of R version 4.0.3, were used to investigate the predictors of OPP exposure [42,43,44]. The parameters for the final models automatically calculated using the h2o package are represented in Supplementary Table S6. For these analyses, the molar sum of DAP (DMP + DMTP + DMDTP + DEP + DETP), dimethyl DAP (DMs: DMP + DMTP + DMDTP) and diethyl DAP (DEs: DEP + DETP) concentrations (µmol/g-creatinine) were calculated. Dwelling information (Table S6) was also included in these models. From the final dataset, 90% of data was randomly selected to build models and the remaining 10% of data was used to validate the models. The process was repeated ten times (10-fold cross-validation).

### 2.8. Cumulative Risk Assessment

The cumulative dose equivalent was calculated to assess the risk of exposure to OPPs referring to the U.S. EPA’s guidelines [25]. The chemical mixture approach was conducted using the following Formulas (2)–(5):

(2) $${µ\mathrm{Mol}}_{\mathrm{DM}}=\left(\frac{C_{\mathrm{DMP}}}{{\mathrm{MW}}_{\mathrm{DMP}}}+\frac{C_{\mathrm{DMTP}}}{{\mathrm{MW}}_{\mathrm{DMTP}}}+\frac{C_{\mathrm{DMDTP}}}{{\mathrm{MW}}_{\mathrm{DMDTP}}}\right)\_\left(\frac{{\mathrm{Cr}}_{\mathrm{ref}}}{{\mathrm{Cr}}_{\mathrm{conc}}}\right)$$

(3) $${µ\mathrm{Mol}}_{\mathrm{DE}}=\left(\frac{C_{\mathrm{DEP}}}{{\mathrm{MW}}_{\mathrm{DEP}}}+\frac{C_{\mathrm{DETP}}}{{\mathrm{MW}}_{\mathrm{DETP}}}\right)\_\left(\frac{{\mathrm{Cr}}_{\mathrm{ref}}}{{\mathrm{Cr}}_{\mathrm{conc}}}\right)$$

(4) $${\mathrm{RPF}}_{i}=\frac{{\mathrm{Relevent}\;\mathrm{dose}}_{\;\mathrm{reference}}}{{\mathrm{Relevent}\;\mathrm{dose}}_{\;\mathrm{chemical}\;i}}$$

(5) $$D_{\mathrm{cum}}=\frac{{µ\mathrm{Mol}}_{\mathrm{DM}}\sum_{i=1}^{8}P_{i}{\mathrm{MW}}_{i}{\mathrm{RPF}}_{i}}{\mathrm{BW}}+\frac{{µ\mathrm{Mol}}_{\mathrm{DE}}\sum_{i=1}^{8}P_{i}{\mathrm{MW}}_{i}{\mathrm{RPF}}_{i}}{\mathrm{BM}}$$

where D_cum_ is the cumulative dose equivalent (µg/kg/day), µMol_DM_ and µMol_DE_ are the total micromoles of DMs and DEs, respectively, excreted over a period of 24 h, Cr_ref_ represents mean daily urinary creatinine excretion of Japanese pregnant women (1050 µg/day) [45], Cr_conc_ is the urinary creatinine concentration (mg/L), C*_i (i = DMP, DMTP, DMDTP, DEP or DETP)_* is the urinary concentration of each OPP metabolite, P*_i_* is the weighted-average proportion of the estimate of OPP release in Japan from 2011 to 2014 (fenitrothion, methidathion, malathion, trichlorfon, dimethoate and pirimiphosmethyl for DMs and diazinon and chlorpyrifos for DEs; Supplementary Table S7) [12], which was calculated using sum of the amount of OPP release in each year multiplying by the ratio of participants sampled in each year, MW*_i_* is the molecular weight of each OPP, RFP is the RPF of the OPP (i.e., the ratio of the BMDL_10_ of chemical *i* to the BMDL_10_ of the reference chemical, i.e., fenitrothion [24]) and BW is the body weight of the participant (kg). Inhibition of brain cholinesterase activity in rats was used as the BMR [46]. The RFPs were calculated using BMDL_10_ of each OPP relative to the NOAEL of fenitrothion [47] (Table S7). A margin of exposure (MOE) of 100 was applied to account for animal-to-human extrapolation.

## 3. Results

### 3.1. Method Performance

The ten-point calibration curve had a coefficient of determination (R^2^) higher than 0.990. The reproducibility for DMP, DMTP, DMDTP, DEP, DETP and DEDTP was 5.1%, 9.1%, 8.0%, 3.7%, 9.6% and 7.8%, respectively. The intermediate precision for DMP, DMTP, DMDTP, DEP, DETP and DEDTP was 4.6%, 6.1%, 5.8%, 4.1%, 6.1% and 5.8%, respectively. The mean concentrations of DMP, DMTP, DMDTP, DEP, DETP and DEDTP in G-EQUAS 61 (sample A/sample B) were 3.42/108, 4.74/63.1, 2.11/8.30, 11.6/34.1, 17.5/63.8 and 0.270/1.82 ng/mL, respectively, which were all within the corresponding tolerance ranges. Agreements of duplicate measurements were 0.0–29.0%, 0.0–29.0%, 0.5–21.1%, 0.2–28.0% and 0.6–27.4% for DMP, DMTP, DMDTP, DEP and DETP, respectively.

### 3.2. Concentrations of DAPs in Maternal Urine Samples

The proportions of samples in which the concentrations of DMP, DMTP, DMDTP, DEP, DETP and DEDTP exceeded the MRLs were 80.8%, 80.0%, 16.1%, 80.2%, 22.9% and 0.02%, respectively (Table 1). The creatinine-normalised median concentrations (interquartile range—IQR) of DMP, DMTP and DEP were 3.53 (1.91–6.78), 4.09 (1.66–10.8) and 3.28 (1.88–5.98) µg/g-creatinine, respectively (Table 1). Meanwhile, the specific gravity-normalised median concentrations of DMP, DMTP and DEP were 3.22 (1.69–6.00), 3.66 (1.53–9.66) and 3.00 (1.68–5.32) ng/mL, respectively (Supplementary Table S8).

### 3.3. Predictors of DAPs in Maternal Urine Samples

**Figure 1:**
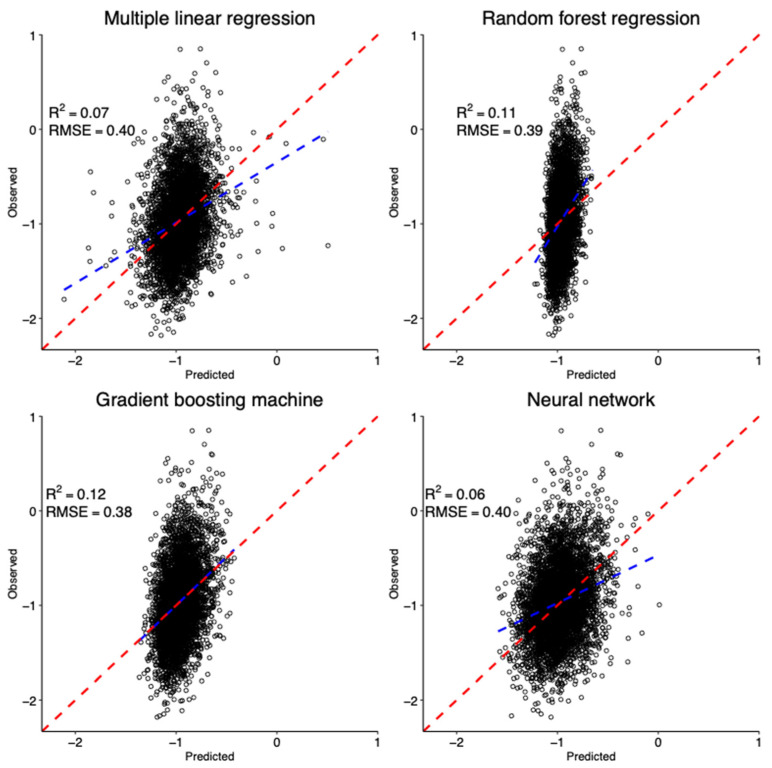
Ten-fold cross-validation of the multiple linear regression, random forest regression, gradient boosting machine and neural network models for urinary DAP concentrations. The blue dotted lines represent the regression lines of the ordinary least square model between predicted and observed concentrations. The red dotted lines have a slope of 1. R^2^, coefficient of determinant; RMSE, root mean square error.

RFR and GBM models presented the highest ten-fold cross-validation coefficients of determination (*R*^2^); however, the *R*^2^ values were <0.15 for all four models (Figure 1, Supplementary Figures S4 and S5).

The month of urine sampling, consumption of apple and maternal BMI were important factors for predicting urinary DAP concentrations (Supplementary Figure S6), similar to the results for DMs and DEs. Consumption of tomato was also of high importance for the prediction of DMs and DEs (Supplementary Figures S7 and S8).

### 3.4. Cumulative Risk Assessment

Table 2 summarises the estimated cumulative OPP dose equivalents. The median (range) estimated cumulative dose equivalents of DAPs, DMs and DEs were 0.45 (0.011–48.7), 0.40 (0.0042–47.8) and 0.035 (0.0011–5.50) µg/kg/day, respectively. Among 4456 participants, 16 (0.34%) study participants’ dose failed to attain an MOE of 100 relative to the BMDL_10_ of the reference chemical (fenitrothion, 13.0 µg/kg weight/day).

## 4. Discussion

This study found that urinary DAP concentrations of pregnant women were lower than in previous studies [3,6,48,49,50,51,52]. The month of urine sampling, consumption of apple and maternal BMI were the main predictors of urinary OPP metabolites, similar to previous studies [10,11]. However, the model performance was poor according to cross-validation, indicating that further information should be collected to investigate predictors of urinary DAP concentrations. We estimated that 1.8% of participants exceeded the BMDL_10_ of the reference chemical from estimation of the cumulative dose equivalents of OPPs.

### 4.1. Concentrations of DAPs in Maternal Urine Samples

To the best of our knowledge, this is the first study to investigate urinary DAP concentrations of Japanese pregnant women on this scale. Urinary DAP concentrations were comparable with those in another Japanese study [21] and in previous studies from other countries [3,6,48,49,50,51,52]. In this study, strict QC measures were employed such as reproducibility, repeatability (intermediate precision), blank test, linearity of the calibration curves, duplicated measurements, recovery of IS samples, target ion/qualifier ion ratio and external QC. This made our reporting limits of some DAPs a magnitude higher than in the previous studies in general. The repeatability of the MRL concentrations was less than 3% relative standard deviation (RSD). Duplicate measurement precision was 0.0–29.0% RSD. This indicates that the overall uncertainty of our measurements was ≤30%.

### 4.2. Predictors of DAPs in Maternal Urine Samples

We investigated the predictors using a conventional multiple linear regression model and three machine learning models. All the models performed poorly according to the ten-fold cross-validation (Figure 1, Figures S4 and S5). This indicates that information collected in JECS was insufficient to predict urinary DAP concentrations. For example, DAPs can be formed naturally in food items [53,54], which was not considered in this study. Some previous studies of pregnant women reported that OPP exposure is associated with the season, maternal BMI and intake of certain food items, such as vegetables, fruits, beans and bread [10,11]. However, these studies did not present the results of cross-validation or good-of-fitness of the models. Although the models had low *R*^2^ values in this study, the month of urine sampling, consumption of apple and maternal BMI were of high importance (Figure S6–S8), similar to the previous studies [10,11]. We do not know the mechanism underlying the relationship between urinary DAP concentrations and maternal BMI; however, the relationship of urinary DAP concentrations with the month of urine sampling and consumption of apple might be related to the use of OPPs on apples. In Japan, OPPs are one of the pesticides used for fruits including apples and are mainly sprayed from May to September [55].

### 4.3. Cumulative Risk Assessment

In this study, 0.36% of participants had doses that exceeded the BMDL_10_/MOE (DAP, median (IQR); 15.5 (14.3–21.0) µg/kg/day), which was two orders of magnitude lower than in a previous study of pregnant women living in an agricultural community [25]. However, it cannot be compared simply because the OPPs included in the current study differ from those included in the previous study. According to Castorina et al. (2003) [25], cumulative dose estimates vary depending on selection of the reference chemical. The reference chemical used in this study was fenitrothion, which has the maximum release in Japan. JECS is a nationally representative cohort; thus, the results of this study can be extrapolated to all pregnant Japanese women. The major adverse effect used to determine BMDL_10_ for OPPs was AChE inhibition in red blood cells or the brain. However, OPPs can be developmental neurotoxicants [1,2]. These effects should be evaluated in the JECS cohort.

### 4.4. Limitations

There are some limitations of this study. The urinary DAP concentrations were measured in spot urine samples. Urinary metabolites of OPPs, i.e., DAPs, have short half-lives ranging from 2 to 15.5 h [17,18]. One study on Japanese pregnant women reported intra-class correlation coefficients (ICCs) to be 0.42–0.55 [21], while studies conducted in other countries provided −0.01–0.52 [51,56,57,58]. According to the rule of thumb, an ICC of 0.4 is categorised as “moderate” [59]. Our data may result in some misclassifications of OPP exposure; thus, the data should be used with caution. One of the reasons why our models performed poorly in predicting urinary DAP concentrations could be missing information about some food items that could carry OPPs and direct (e.g., occupational) or indirect (e.g., vicinity to farmlands) OPP use. In addition, the intake of DAPs derived from degradation of OPPs in food stuff was not taken into account, which might lead to the overestimation of DAP exposure.

In terms of cumulative risk assessment, there are the following uncertainties: (1) the individual volumes of 24 h urine were estimated using the reference creatinine excretion relative to participants’ urine creatinine concentrations, (2) the proportions of OPP emission were used to calculate cumulative daily intake instead of personal OPP use of individual participants, (3) only half the OPPs used in Japan are metabolised into DAPs and thus were included in the risk assessment and (4) it was assumed that 100% of OPPs taken up were metabolised into DAPs and this might have resulted in underestimation of OPP exposure.

## 5. Conclusions

Median urinary concentrations of DMP, DMTP and DEP were 3.53, 4.09 and 3.28 µg/g-creatinine, respectively, which were comparable with those in previous studies of pregnant women. Even though the variables selected using machine learning models were similar to those reported in previous studies, the prediction models performed poorly. This indicates that a further study is needed to include more information about the intake of foods that likely contain OPPs or DAPs and investigate other unknown factors. Daily intake of OPPs exceeded the BMDL_10_ of the reference chemical in 0.36% of participants based on cumulative risk assessment. Considering that this study is nationally representative and OPP exposure may have been overestimated in this study, Japanese pregnant women are not at risk by the cumulative OPP exposure.

## Figures and Tables

**Table 1:** Urinary DAP concentrations (*n* = 4575).

Statistics	Crude (ng/mL)						Creatinine-Normalised (µg/g-Creatinine)					
	DMP	DMTP	DMDTP	DEP	DETP	DEDTP ^c^	DMP	DMTP	DMDTP	DEP	DETP	DEDTP
DR (%)	80.8	80.0	16.1	81.2	22.9	0.02	80.8	80.0	16.1	81.2	22.9	0.02
Mean	5.69	11.3	-	5.12	-	-	5.79	11.8	-	5.32	-	-
SD	10.4	45.6	-	8.97	-	-	8.07	31.5	-	9.54	-	-
Min	<MRL ^a^	<MRL ^a^	<MRL ^a^	<MRL ^a^	<MRL ^b^	-	<MRL					-
25th	1.33	1.32	-	1.32	-	-	1.91	1.66	-	1.88	-	-
50th	2.93	3.29	-	2.78	-	-	3.53	4.09	-	3.28	-	-
75th	6.16	9.38	-	5.47	-	-	6.78	10.8	-	5.98	-	-
95th	18.6	40.2	2.94	16.1	4.50	-	17.8	44.5	3.22	15.3	5.15	-
Max	385	1640	95.4	213	319	34.9	226	891	50.4	380	681	126

^a^ 0.997 ng/mL in urine samples, ^b^ 1.2 or 1.3 ng/mL in urine samples, ^c^ only one sample had a DEDTP concentration above the MRL. Mean and SD were calculated after imputation. DMP, dimethylphosphate; DMTP, dimethylthiophosphate; DMDTP, dimethyldithiophosphate; DEP, diethylphosphate; DETP, diethylthiophosphate; DEDTP, diethyldithiophosphate; DR, detection rate; MRL, minimum reporting level.

**Table 2:** Estimated cumulative OPP dose equivalents (µg/kg/day, *n* = 4456).

Statistics	DAPs	DMs	DEs
Mean	0.88	0.82	0.058
SD	1.72	1.69	0.13
GM	0.47	0.42	0.036
GSD	2.82	2.98	2.52
Min	0.011	0.0042	0.0011
25th	0.23	0.20	0.019
50th	0.45	0.40	0.035
75th	0.92	0.84	0.063
95th	2.90	2.78	0.17
Max	48.7	47.8	5.50
>BMDL_10_/100 ^a^ —*n*, (%)	16 (0.36)	16 (0.36)	0 (0)

^a^ The reference chemical (fenitrothion) = 13.0 µg/kg weight/day. BMDL_10_, lower 95% confidence limit of the benchmark dose_10_; SD, standard deviation; GM, geometric mean; GSD, geometric standard deviation; DAPs, dialkylphosphates (DMP + DMTP + DMDTP + DEP + DETP); DMs, dimethylphosphate metabolites (DMP + DMTP + DMDTP); DEs, diethylphosphate metabolites (DEP + DETP).

## Data Availability

Data are unsuitable for public deposition due to ethical restrictions and the legal framework of Japan. It is prohibited by the Act on the Protection of Personal Information (Act No. 57 of 30 May 2003, amendment on 9 September 2015) to publicly deposit data containing personal information. Ethical Guidelines for Medical and Health Research Involving Human Subjects enforced by the Japan Ministry of Education, Culture, Sports, Science and Technology and the Ministry of Health, Labour and Welfare also restricts the open sharing of the epidemiologic data. All inquiries about access to data should be sent to: jecs-en@nies.go.jp. The person responsible for handling enquiries sent to this e-mail address is Dr Shoji F. Nakayama, JECS Programme Office, National Institute for Environmental Studies.

## Supplementary Materials

The following are available online at https://www.mdpi.com/article/10.3390/ijerph18115929/s1, Table S1: LC gradient conditions; Table S2: Mass transitions monitored; Table S3: Ion source and collision cell conditions; Table S4: Range of calibration curve; Table S5: Parameters of the three machine learning approaches in the final models; Table S6: Characteristics data for the participants of the Japan Environment and Children’s Study; Table S7: Relevant doses (BMDL_10_ or NOAEL) and relative potency factors; Table S8: Urinary DAP concentrations (*n* = 4575 specific gravity-normalised, ng/mL); Figure S1: Flow chart of the study participant selection; Figure S2: Sample treatment for measurement of urinary DAPs; Figure S3: Column switching system for the measurement of urinary DMTP, DETP, DMDTP and DEDTP concentrations with LC-MS/MS; Figure S4: Ten-fold cross-validation of the multiple linear regression, random forest regression, gradient boosting machine and neural network models for DM concentrations. The blue dotted lines represent the regression lines of the ordinary least square model between observed and predicted concentrations. The red dotted lines have a slope of 1; Figure S5: Ten-fold cross-validation of the multiple linear regression, random forest regression, gradient boosting machine and neural network models for DE concentrations. The blue dotted lines represent the regression lines of the ordinary least square model between observed and predicted concentrations. The red dotted lines have a slope of 1; Figure S6: Variable importance of the important features selected by gradient boosting machine for DAPs. The *x*-axis represents the importance value of each variable; Figure S7: Variable importance of the important features selected by gradient boosting machine for DMs. The *x*-axis represents the importance value of each variable; Figure S8: Variable importance of the important features selected by gradient boosting machine for DEs. The *x*-axis represents the importance value of each variable.

## Abbreviations

AChE	acetylcholinesterase
BMD	benchmark dose
BMD_10_	10% onset of toxicity is used for general toxicity as the BMR
BMDL	BMD lower confidence limit
BMI	body mass index
BMR	bench-mark response
DAPs	dialkylphosphates
DEDTP	diethyldithiophosphate
DEP	diethylphosphate
DEs	diethyl DAP (DEP + DETP)
DETP	diethylthiophosphate
DMDTP	dimethyldithio-phosphate
DMP	dimethylphosphate
DMs	dimethyl DAP (DMP + DMTP + DMDTP)
DMTP	dimethylthiophosphate
DR	detection rate
G-EQUAS	German External Quality Assessment Scheme
GBM	gradient boosting machine
IQR	interquartile range
LC-MS/MS	liquid chromatography-tandem mass spectrometer
LCMRL	lowest concentration minimum reporting level
MICE	multiple imputation by chained equations
MOE	margin of exposure
MRL	minimum reporting level
MW	molecular weight
NOAEL	no observed adverse effect level
OPPs	organophosphate pesticides
QC	quality control
QRLIC	quantile regression approach for left-censored missing
RFPs	elative potency factors
RFR	random forest regression
RSD	relative standard deviation.
SD	standard deviation
U.S. EPA	The U.S. Environmental Protection Agency
