# Supplementary material for: Urinary Metabolites of Organophosphate Pesticides among Pregnant Women Participating in the Japan Environment and Children’s Study (JECS)
Source: Int J Environ Res Public Health. 2021 May 31;18(11):5929. doi: 10.3390/ijerph18115929 (PMC8199379; doi:10.3390/ijerph18115929)
Supplement: Supplementary file 1 [file ijerph-18-05929-s001.zip › ijerph-1232301-supplementary.pdf]

Supplementary Information

Urinary metabolites of organophosphate pesticides among pregnant women participating in the Japan Environment and Children's Study (JECS)

Yukiko Nishihama[1], Shoji F. Nakayama[1], Tomohiko Isobe[1], Chau-Ren Jung[1,2], Miyuki Iwai-Shimada[1], Yayoi Kobayashi[1], Takehiro Michikawa[1,3], Makiko Sekiyama[1], Yu Taniguchi[1] and Shin Yamazaki[1] on behalf of the Japan Environment and Children's Study Group

1. Japan Environment and Children's Study Programme Office, Health and Environmental Risk Division, National Institute for Environmental Studies, Tsukuba, Japan
2. Department of Public Health, College of Public Health, China Medical University
3. Department of Environmental and Occupational Health, School of Medicine, Toho University, Tokyo, Japan

Corresponding author:

Shoji F. Nakayama

Japan Environment and Children's Study Programme Office, Health and Environmental Risk Division, National Institute for Environmental Studies 16-2 Onogawa, Tsukuba, Ibaraki 305-8506, Japan

Phone: +81 29 850 2786

E-mail: fabre@nies.go.jp

Table S1. LC gradient conditions.

DMP, DEP[a]			
Time (min)	Mobile phase A (%)		Mobile phase B (%)
0.01	4		96
3.50	4		96
6.00	10		90
6.01	20		80
8.00	20		80
9.00	4		96
13.0	4		96
Flow rate	0.4 ml/min		
Injection volume	2 μl		
Solvent for needle wash	Acetonitrile/water = 7/3 (v/v)		
Temperature of autosampler	4°C		
DMTP, DMDTP, DETP, DEDTP[b]			
Time(min)	Mobile phase A (%)	Mobile phase B (%)	Mobile phase C (%)
0.01	100	0	100
1.00	100	0	100
6.00	30	70	100
17.0	30	70	100
18.0	100	0	100
22.0	100	0	100
Flow rate	0.3 ml/min		
Injection volume	1 μl		
Solvent for needle wash	Acetonitrile/water/formic acid = 80/20/0.005 (v/v/v)		
Temperature of autosampler	4°C		

[a]Mobile phase A: 50 mM ammonium acetic acid, mobile phase B: acetonitrile. [b]Mobile phase A: acetonitrile/water/formic acid = 80/20/0.005 (v/v/v), mobile phase B: acetonitrile with 5 mM ammonium acetic acid/water/formic acid = 95/5/0.05 (v/v/v), mobile phase C: acetonitrile with 5 mM ammonium acetic acid/water = 95/5 (v/v).

Table S2. Mass transitions monitored

Compound	Target ion (m/z)		Qualifier ion (m/z)		Declustering potential (V)		Collision energy (eV)		Ionization mode
	Precursor	Product	Precursor	Product	Target ion	Qualifier ion	Target ion	Qualifier ion	
DMP	125.0	63.0	125.0	111.0	−40	−40	−22	−22	Negative
DMTP	141.0	96.0	141.0	63.0	−35	−35	−28	−43	
DMDTP	157.0	142.0	157.0	112.0	−60	−60	−22	−38	
DEP	153.0	125.0	153.0	79.0	−32	−32	−14	−25	
DETP	169.0	95.0	169.0	141.0	−35	−35	−25	−16	
DEDTP	185.0	111.0	185.0	79.0	−40	−40	−25	−59	
DMP-d6	131.0	62.8	-	-	−20	-	−23	-	
DMTP-d6	147.0	129.0	-	-	−35	-	−23	-	
DMDTP-d6	163.0	145.0	-	-	−60	-	−23	-	
DEP-d10	163.0	131.0	-	-	−35	-	−17	-	
DETP-d10	179.0	147.0	-	-	−35	-	−19	-	
DEDTP-d10	195.0	111.0	-	-	−50	-	−25	-	

Table S3. Ion source and collision cell conditions.

Parameter	Setting
IonSpray voltage (V)	−4500
Heating gas temperature (°C)	500
Nebulizer gas (psi)	50
Heating gas (psi)	60
Curtain gas flow (psi)	20
Collision gas pressure	11

Table S4. Range of calibration curve

Native	Concentration in urine sample (ng/ml)									Internal standard	Concentration (ng/ml)
DMT, DMTP, DMDTP,DEP, DETP, DEDTP	0	1.0	2.0	4.0	10	20	40	80	100	DMP-d6, DMTP-d6, DMDTP-d6, DEP-d10, DETP-d10, DEDTP-d10	50

Table S5. Characteristics data for the participants of the Japan Environment and Children's Study

Variables (unit)	Summary[a]	N
Regional centre		4,456
Hokkaido	366 (8.2)	
Miyagi	396 (8.9)	
Fukushima	570 (12.8)	
Chiba	262 (5.9)	
Kanagawa	284 (6.4)	
Koshin	328 (7.4)	
Toyama	248 (5.6)	
Aichi	259 (5.8)	
Kyoto	174 (3.9)	
Osaka	331 (7.4)	
Hyogo	221 (5.0)	
Tottori	124 (2.8)	
Kochi	285 (6.4)	
Fukuoka	346 (7.8)	
South Kyusyu/Okinawa	262 (5.9)	
Month		4,456
Jan.	447 (10.0)	
Feb.	458 (10.3)	
Mar.	526 (11.8)	
Apr.	455 (10.2)	
May	347 (7.8)	
June	254 (5.7)	
July	265 (5.9)	
Aug.	209 (4.7)	
Sept.	236 (5.3)	
Oct.	370 (8.3)	
Nov.	414 (9.3)	
Dec.	475 (10.7)	4,456
Maternal age (years old)	32 (28, 35)	4,456
Maternal BMI (kg/m^2)	20.7 (19.1, 22.5)	4,456
Gestational week (weeks)	15.7 (13.6, 17.9)	4,456
Urinary creatinine concentration (mg/dl)	87.6 (51.9, 131)	4,456
eGFR (ml/min/1.73 m^2)[b]	129 (116, 145)	4,434
Maternal smoking		4,456
No	4,280 (96.1)	
Yes	154 (3.5)	
Maternal alcohol consumption		4,437
No	3,956 (88.8)	
Yes	481 (10.8)	
Frequency of the occupational use of insecticide during the firsttrimester		3,991
No	3,791 (85.1)	
1–3 times a month	174 (3.9)	
1–6 times a week	26 (0.6)	
Everyday	0 (0.0)	
Frequency of the occupational use of herbicide during the firsttrimester		3,987
No	3,956 (88.8)	
1–3 times a month	29 (0.7)	
1–6 times a week	2 (0.0)	
Every day	0 (0.0)	
Frequency of the occupational use of other agrichemicals during the first trimester		3,926
No	3,920 (88.0)	
1–3 times a month	5 (0.1)	
1–6 times a week	0 (0.0)	
Everyday	1 (0.0)	
Household income		4,257
< 2 million Japanese yen (~18,181 USD; 1 USD 110 yen)	187 (4.2)	
2 to < 4 million yen (~36,363 USD)	1,372 (30.8)	
4 to < 6 million yen (~54,545 USD)	1,466 (32.9)	
6 to < 8 million yen (~72,727 USD)	720 (16.2)	
8 to < 10 million yen (~90,909 USD)	321 (7.2)	
10 to < 12 million yen (~109,090 USD)	104 (2.3)	
12 to < 15 million yen (~136,363 USD)	36 (0.8)	
15 to < 20 million yen (~181,818 USD)	32 (0.7)	
≥ 20 million yen	19 (0.4)	
Education		4,433
Junior high school	164 (3.7)	
High school	1,207 (27.1)	
Higher professional school	80 (1.8)	
Technical school	1,050 (23.6)	

Junior college	782 (17.5)	
University	1,080 (24.2)	
Graduate school	70 (1.6)	
Use of a moth repellent for clothes in the closet during the second orthird trimester		4,434
Never	1,763 (39.6)	
Yes, sometimes	1,648 (37.0)	
Yes, continuously	1,023 (23.0)	
Use of smoke insecticide indoors during the second or third trimester		4,437
No	4,167 (93.5)	
Yes	270 (6.1)	
Frequency of the occupational use of insecticide during the second orthird trimester		4,317
No	3,949 (88.6)	
1–3 times a month	327 (7.3)	
1–6 times a week	36 (0.8)	
Everyday	5 (0.1)	
Frequency of the occupational use of herbicide during the second orthird trimester		4,268
No	4,211 (94.5)	
1–3 times a month	53 (1.2)	
1–6 times a week	3 (0.1)	
Everyday	1 (0.0)	
Use of a water purifier in a water faucet during the second or thirdtrimester		4,431
Yes	1,338 (30.0)	
No	3,046 (68.4)	
Other	47 (1.1)	
Use of a spray insecticide indoors during the second or third trimester		4,437
No	3,094 (69.4)	
Less than once a month	833 (18.7)	
1–3 times a month	255 (5.7)	
Once a week	84 (1.9)	
A few times a week	153 (3.4)	
Everyday	18 (0.4)	
Use of a mosquito coil or electric mosquito repellent mat during thesecond or third trimester		4,436
No	3,144 (70.6)	
Less than once a month	237 (5.3)	
1–3 times a month	170 (3.8)	
Once a week	99 (2.2)	
A few times a week	423 (9.5)	
Everyday	363 (8.1)	
Use of a liquid insecticide for maggot and mosquito larva during thesecond or third trimester		4,442
No	4,418 (99.1)	
Less than once a month	16 (0.4)	
1–3 times a month	5 (0.1)	
Once a week	0 (0.0)	
A few times a week	3 (0.1)	
Everyday	0 (0.0)	
Use of an herbicide or a gardening pesticide in a garden, balcony orfarm during the second or third trimester		4,426
No	4,020 (90.2)	
Less than once a month	332 (7.5)	
1–3 times per month	56 (1.3)	
Once a week	12 (0.3)	
A few times a week	4 (0.1)	
Everyday	2 (0.0)	
Food items (g/day)		4,455
Sake	0.00 (0.00, 0.00)	
Shochu	0.00 (0.00, 0.00)	
Beer	0.00 (0.00, 0.00)	
Whiskey	0.00 (0.00, 0.00)	
Wine	0.00 (0.00, 0.00)	
Rice	280 (220, 420)	
Brown rice	0.00 (0.00, 0.00)	
Wheat	0.00 (0.00, 0.00)	
Other crops	0.00 (0.00, 0.00)	
Miso soup	75.0 (32.1, 150)	
Beef steak	0.00 (0.00, 0.00)	
Grilled beef	3.33 (0.00, 6.67)	
Stir-fried beef	4.00 (0.00, 6.43)	
Stewed beef	1.67 (0.00, 3.33)	
Stir-fried pork	12.9 (4.00, 12.9)	
Fried pork	3.33 (0.00, 6.67)	
Stewed pork (e.g., curry)	3.33 (0.00, 5.00)	
Stewed pork	0.00 (0.00, 0.00)	
Pork soup	1.33 (0.00, 2.67)	
Liver of pork	0.00 (0.00, 0.00)	
Grilled chicken	4.67 (0.00, 7.00)	
Stir-fried chicken	4.00 (0.00, 6.00)	
Stewed chicken	3.33 (0.00, 3.33)	
Fried chicken	3.33 (3.33, 5.00)	
Liver of chicken	0.00 (0.00, 0.00)	

Ham	1.00 (0.00, 3.21)
Sausage	6.43 (2.00, 7.50)
Bacon	1.33 (0.00, 4.29)
Canned meet	0.00 (0.00, 0.00)
Cow's milk, low-fat	0.00 (0.00, 0.00)
Cow's milk	42.9 (0.00, 100)
Egg	25.0 (10.7, 39.3)
Cheese	4.29 (1.33, 4.29)
Yogurt	25.7 (8.00, 90.0)
Salted fish	4.67 (0.00, 4.67)
Dried fish	0.00 (0.00, 3.33)
Canned tuna	1.33 (0.00, 1.33)
Salmon	4.67 (0.00, 4.67)
Tuna	2.00 (0.00, 4.00)
Yellowtail	0.00 (0.00, 4.00)
Cod plaice	0.00 (0.00, 2.67)
Snapper	0.00 (0.00, 0.00)
Trevally sardine	0.00 (0.00, 5.33)
Saury mackerel	5.33 (0.00, 5.33)
Dried pilchard	0.00 (0.00, 0.67)
Fish egg	0.00 (0.00, 1.33)
Eel	0.00 (0.00, 0.00)
Squid	0.00 (0.00, 3.33)
Octopus	0.00 (0.00, 3.33)
Shrimp	2.67 (0.00, 2.67)
Clam	0.00 (0.00, 1.33)
River snail	0.00 (0.00, 0.00)
Chikuwa	1.33 (0.00, 1.33)
Kamaboko	0.00 (0.00, 1.33)
Satsuma-age	0.00 (0.00, 1.33)
Carrot	9.13 (7.82, 18.3)
Spinach	6.64 (2.07, 9.96)
Pumpkin	2.56 (0.85, 5.49)
Cabbage	8.87 (5.91, 20.7)
Daikon	9.09 (4.24, 13.6)
Takuan	0.00 (0.00, 1.12)
Pickled green	0.00 (0.00, 1.16)
Pickled plum	0.48 (0.00, 1.54)
Pickled nappa	0.54 (0.00, 3.47)
Pickled cucumber	0.72 (0.00, 2.31)
Pickled aubergine	0.00 (0.00, 0.68)
Pickled turnip	0.00 (0.00, 0.96)
Bell pepper	1.41 (0.88, 3.30)
Tomato	6.27 (1.95, 14.6)
Allium	2.79 (0.87, 6.50)
Tree onion	0.30 (0.00, 0.96)
Garlic chive	0.67 (0.00, 1.07)
Glebionis coronaria	0.00 (0.00, 1.36)
Komatsuna	0.84 (0.00, 2.70)
Broccoli	2.10 (1.40, 6.75)
Onion	23.8 (10.2, 37.3)
Cucumber	5.11 (1.59, 11.9)
Aubergine	2.12 (0.66, 6.36)
Nappa	3.73 (1.16, 8.70)
Burdock	2.16 (0.00, 6.94)
Mung bean sprout	4.07 (1.27, 6.11)
Green bean	0.00 (0.00, 0.72)
Lettuce	0.94 (0.29, 1.41)
Asparagus	0.53 (0.00, 0.80)
Garlic	0.06 (0.00, 0.19)
Pak choi	0.00 (0.00, 2.43)
Brown mustard	0.00 (0.00, 0.00)
Bitter melon	0.00 (0.00, 1.67)
Swiss chard	0.00 (0.00, 0.00)
Sponge gourd	0.00 (0.00, 0.00)
Mug wort	0.00 (0.00, 0.00)
Papaya	0.00 (0.00, 0.00)
Tangerine	15.3 (4.76, 35.7)

Other citrus fruits	2.45 (0.00, 7.88)
Apple	5.19 (1.73, 13.0)
Persimmon	1.07 (0.00, 3.43)
Strawberry	3.60 (1.20, 9.00)
Grape	2.00 (0.00, 6.43)
Melon	0.00 (0.00, 1.44)
Watermelon	2.16 (0.00, 6.94)
Peach	1.08 (0.00, 3.48)
Nashi (pear)	1.87 (0.00, 6.00)
Kiwi	1.57 (0.00, 2.35)
Pineapple	0.00 (0.00, 5.29)
Banana	3.05 (0.00, 9.80)
Mango	0.00 (0.00, 0.00)
Bread	30.0 (12.9, 60.0)
Udon	26.8 (16.7, 53.6)
Soba	13.3 (0.00, 13.3)
Okinawa soba	0.00 (0.00, 0.00)
Ramen	14.7 (14.7, 47.1)
Pasta	16.7 (16.7, 53.6)
Somen	13.3 (0.00, 13.3)
Mochi	0.00 (0.00, 3.33)
Japanese confections	0.00 (0.00, 4.67)
Cake	4.67 (0.00, 4.67)
Biscuit	1.67 (0.00, 5.36)
Chocolate	2.68 (0.83, 6.25)
Ice cream	5.33 (5.33, 17.1)
Snacks	2.50 (1.67, 8.04)
Rice cracker	1.00 (0.00, 3.21)
Sesame	0.03 (0.00, 0.11)
Peanut	0.00 (0.00, 0.00)
Tofu in miso soup	4.29 (1.33, 10.0)
Tofu	5.00 (5.00, 16.1)
Yushi tofu	0.00 (0.00, 0.00)
Koya tofu	0.00 (0.00, 0.00)
Fried tofu	2.00 (0.00, 4.00)
Deep-fried tofu	0.13 (0.00, 0.43)
Natto	10.7 (3.33, 25.0)
Sweet potato	2.67 (0.00, 4.00)
Potato	10.7 (5.00, 16.1)
Taro	0.00 (0.00, 2.00)
Yam	0.00 (0.00, 3.33)
Konjac	3.33 (0.00, 3.33)
Shiitake	1.33 (0.00, 4.29)
Enoki	2.00 (1.33, 4.29)
Shimeji	4.29 (1.33, 4.29)
Seaweed	4.29 (1.33, 4.29)
Hijiki	1.33 (0.00, 1.33)
Nori	0.13 (0.07, 0.43)
Butter on bread	0.00 (0.00, 0.53)
Margarine on bread	0.53 (0.00, 1.71)
Jam on bread	0.00 (0.00, 0.86)
Honey	0.00 (0.00, 0.47)
Salad dressing	2.14 (0.67, 5.00)
Mayonnaise	1.50 (0.47, 3.50)
Sauce	0.33 (0.17, 1.07)
Ketchup	0.50 (0.33, 1.07)
Japanese mustard	0.00 (0.00, 0.07)
Wasabi	0.03 (0.00, 0.07)
Chili pepper	0.00 (0.00, 0.03)
Ginger	0.33 (0.00, 1.07)
Kinako	0.00 (0.00, 0.00)
Brewed green tea	25.7 (0.00, 94.3)
Bottled green tea	42.9 (0.00, 42.9)
Brewed oolong tea	0.00 (0.00, 0.00)
Bottled oolong tea	0.00 (0.00, 42.9)
Brewed black tea	0.00 (0.00, 25.7)
Bottled black tea	0.00 (0.00, 42.9)
Brewed coffee	0.00 (0.00, 25.7)

Instant coffee	0.00 (0.00, 60.0)	
Bottled coffee	0.00 (0.00, 53.6)	
Tomato juice	0.00 (0.00, 0.00)	
Vegetable juice	0.00 (0.00, 42.9)	
Orange juice	0.00 (0.00, 42.9)	
Apple juice	0.00 (0.00, 42.9)	
Grapefruit juice	0.00 (0.00, 0.00)	
Fruit juice	0.00 (0.00, 42.9)	
Carbonated drink	0.00 (0.00, 75.0)	
Soy milk	0.00 (0.00, 0.00)	
Fermented milk drink	0.00 (0.00, 42.9)	
Tap or well water	0.00 (0.00, 100)	
Bottled water	42.9 (0.00, 200)	
Sweetened black tea	0.00 (0.00, 0.00)	
Tea with milk	0.00 (0.00, 0.00)	
Sweetened coffee	0.00 (0.00, 0.00)	
Coffee with milk	0.00 (0.00, 0.00)	
Salt	2.73 (1.89, 3.89)	
Noodle soup	21.3 (9.05, 47.5)	
Vegetable oil	3.82 (2.25, 7.28)	
Safflower oil	0.00 (0.00, 0.00)	
Corn oil	0.00 (0.00, 0.00)	
Soybean oil	0.00 (0.00, 0.00)	
Canola oil	0.00 (0.00, 3.12)	
Olive oil	0.00 (0.00, 0.00)	
Other oil	0.00 (0.00, 0.00)	
Food groups (g/day)		4,455
Cereal	457 (368, 545)	
Tubers	20.1 (11.7, 33.3)	
Sugar	0.00 (0.00, 0.60)	
Beans	37.5 (19.8, 69.6)	
Nuts	0.00 (0.00, 0.60)	
Vegetables	164 (103, 246)	
Pickles	4.50 (0.80, 12.3)	
Greens	70.5 (37.5, 121)	
Other vegetables	83.3 (53.1, 124)	
Fruits	118 (52.8, 205)	
Mushrooms	8.40 (4.00, 12.9)	
Algae	4.40 (1.50, 8.60)	
Fish	32.0 (18.0, 51.6)	
Meat	61.5 (39.7, 92.1)	
Eggs	25.0 (10.7, 39.3)	
Milk	167 (81.4, 281)	
Oils	9.90 (6.80, 13.7)	
Confections	19.5 (9.80, 33.4)	
Alcohol	0.00 (0.00, 0.00)	
Non-alcohol drink	355 (195, 600)	
Condiment	16.5 (10.8, 23.8)	
Water	157 (42.9, 500)	
Soup	105 (55.9, 172)	

[a] Categorical variables are shown as n (%) and continuous variables are shown as median (IQR). [b] eGFR = 194×serum creatinine concentration$^{-1.094}$×maternal age$^{-0.287}$×0.739 (Matsuo et al., https://doi.org/10.1053/j.ajkd.2008.12.034). The imputed maternal age value was used.

Table S6. Parameters of the three machine learning approaches in the final models

	DAPs	DMs	DEs
Random forest regression			
Number of trees	500	500	500
Depth of trees	19	19	6
m_{try}	48	48	71
Gradient boosting machine			
Number of trees	100	100	500
Depth of trees	4	3	1
Learning rate	0.052	0.065	0.046
Neural network analysis			
Activation function	Tanh	Tanh	Tanh
Learning rate	0.005	0.005	0.005

Table S7. Relevant doses ($BMDL_{10}$ or NOAEL) and relative potency factors

**Pesticide**	**$BMDL_{10}$/100 (mg/kg weight/day)**	**RPF**	**Source**	**P (%)**
Fenitrothion	0.013[a]	1.0 (reference)	JMPR07	40.1
Diazinon	0.0017	7.6	EPA02	30.1
Malathion	0.29	0.045	EPA02	9.7
Chlorpyrifos	0.0022	5.9	EPA02	6.6
Methidathion	2.2	0.0059	EPA02	5.8
Dimethoate	0.029	0.45	EPA02	3.4
Trichlorfon	0.016	0.81	EPA02	2.8
Pirimiphos-methyl	0.013	1.0	EPA02	1.5

[a]The NOAEL for inhibition of brain cholinesterase activity in rats for 13 weeks. $BMDL_{10}$, lower 95% confidence limit of the benchmark $dose_{10}$; NOAEL, no observed adverse effect level; RPF, relative potency factor; P, the proportion of the estimate of OPP release (https://www.env.go.jp/chemi/prtr/result/todokedegai_siryo.html).

Table S8. Urinary DAP concentrations (N = 4,575, specific gravity-normalised, ng/ml)

	**DMP**	**DMPT**	**DMDTP**	**DEP**	**DETP**	**DEDTP[a]**
DR (%)	80.8	80.0	16.1	81.2	22.9	0.02
Mean	5.35	10.8	-	4.85	-	-
SD	8.06	32.4	-	7.83	-	-
Min			<MRL			-
25th	1.69	1.53	-	1.68	-	-
50th	3.22	3.66	-	3.00	-	-
75th	6.00	9.66	-	5.32	-	-
95th	16.4	41.6	2.95	14.1	4.77	-
Max	263	1080	55.8	217	437	77.8

[a]Only one sample had a DEDTP concentration above the MRL. Mean and SD were calculated after imputation. DMP, dimethylphosphate; DMTP, dimethylthiophosphate; DMDTP, dimethyldithiophosphate; DEP, diethylphosphate; DETP, diethylthiophosphate; DEDTP, diethyldithiophosphate; DR, detection rate; MRL, minimum reporting level.

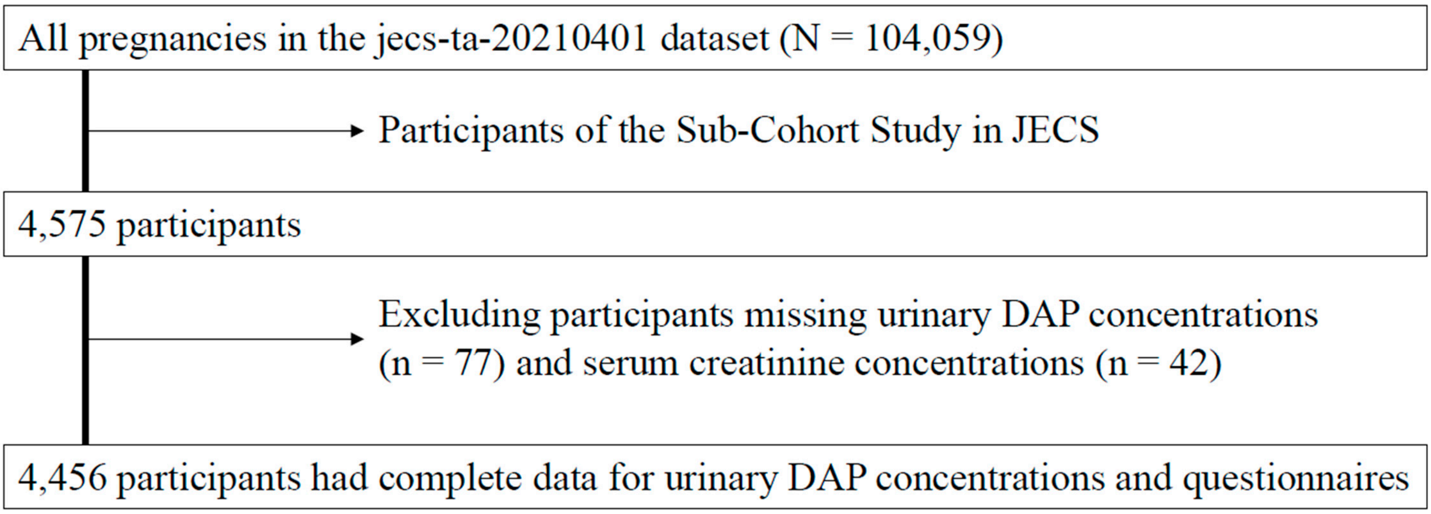

Figure S1. Flow chart of the study participant selection.

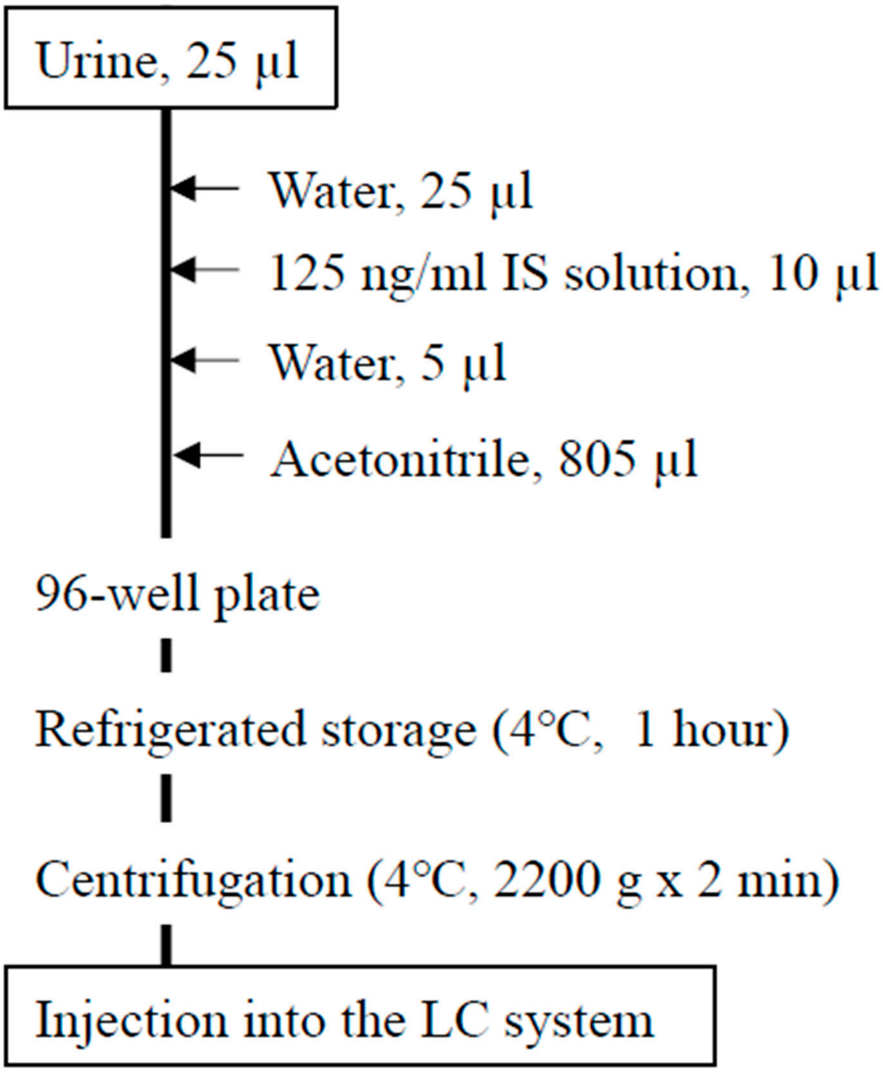

Figure S2. Sample treatment for measurement of urinary DAPs.

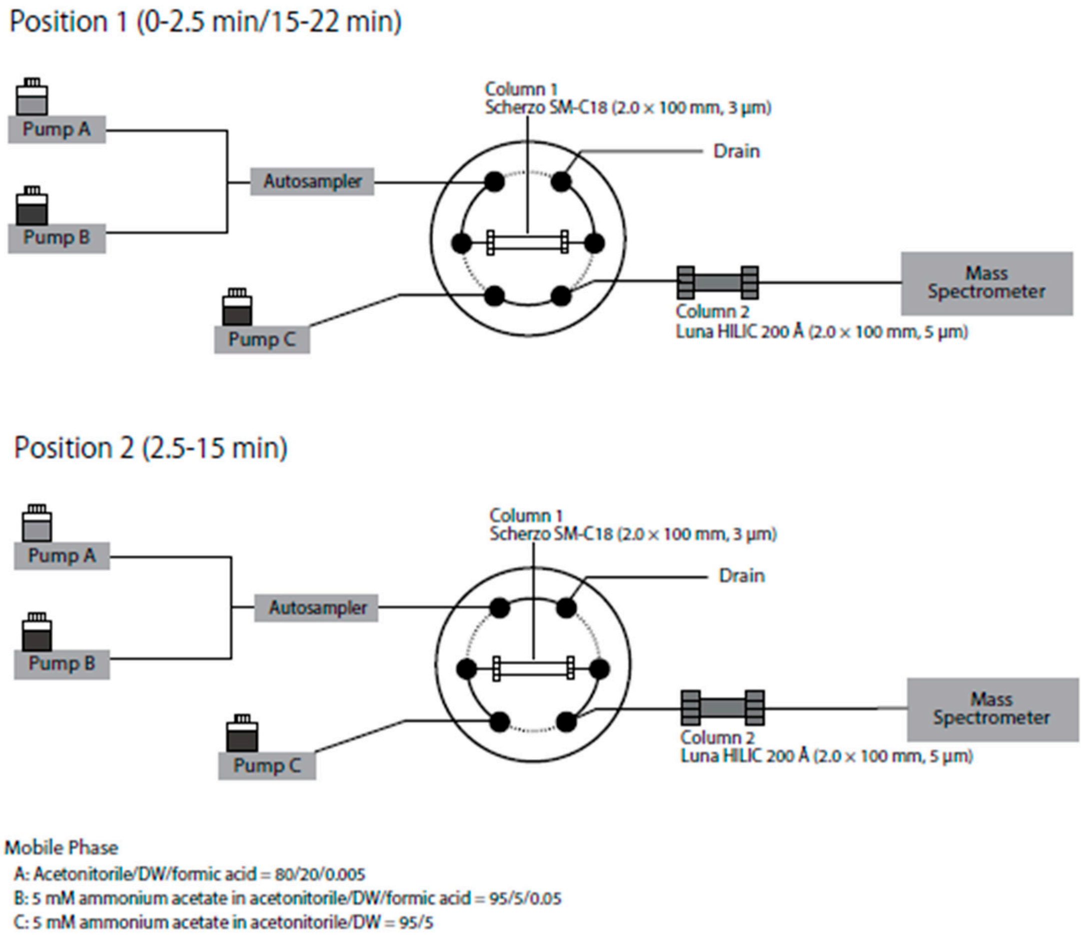

Figure S3. Column switching system for the measurement of urinary DMTP, DETP, DMDTP and DEDTP 72 concentrations with LC-MS/MS.

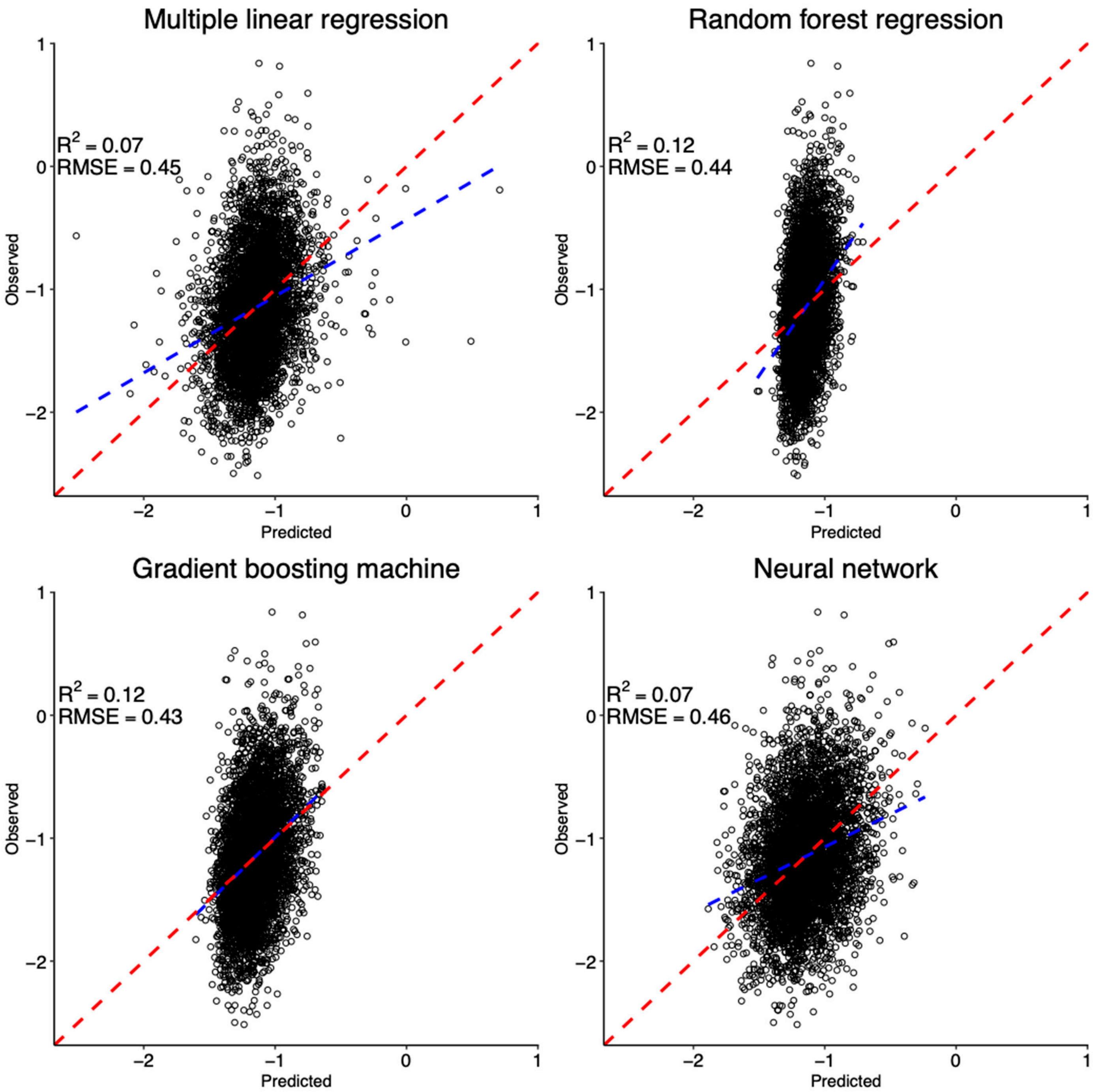

Figure S4. Ten-fold cross-validation of the multiple linear regression, random forest regression, gradient boosting machine and neural network models for DM concentrations. The blue dotted lines represent the regression lines of the ordinary least square model between observed and predicted concentrations. The red dotted lines have a slope of 1.

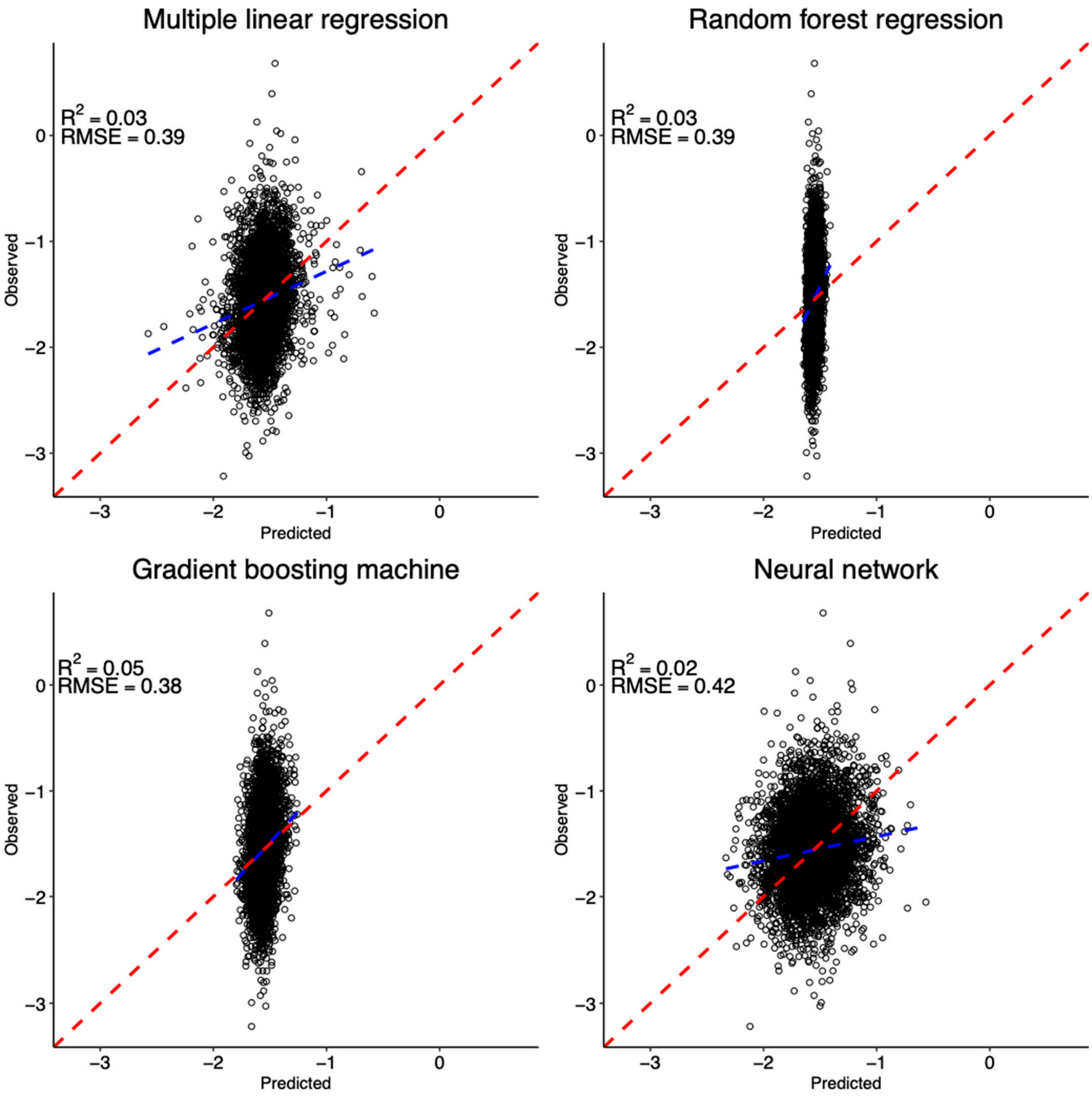

Figure S5. Ten-fold cross-validation of the multiple linear regression, random forest regression, gradient boosting machine and neural network models for DE concentrations. The blue dotted lines represent the regression lines of the ordinary least square model between observed and predicted concentrations. The red dotted lines have a slope of 1.8

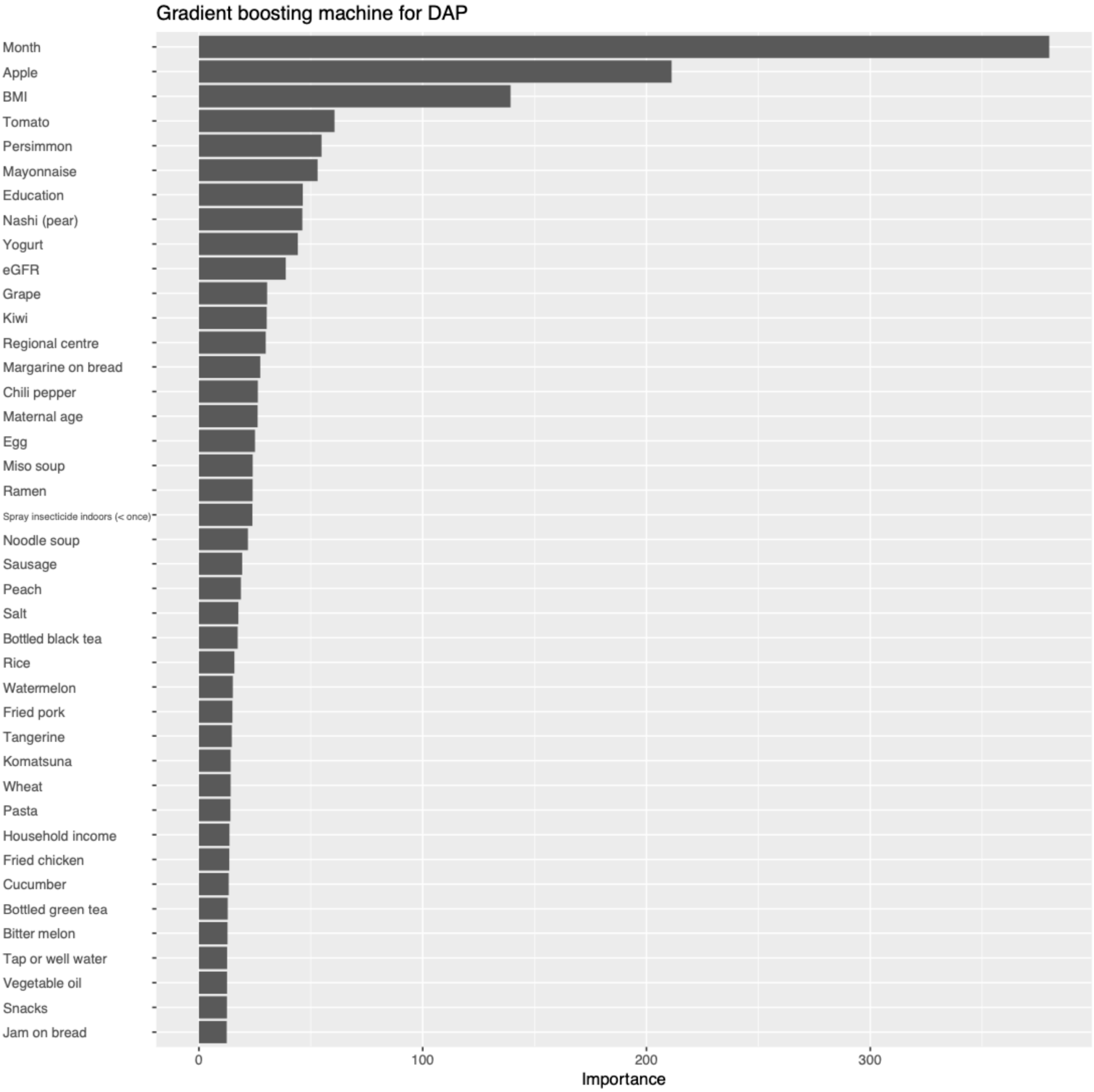

Figure S6. Variable importance of the important features selected by gradient boosting machine for DAPs. The x-axis represents the importance value of each variable.

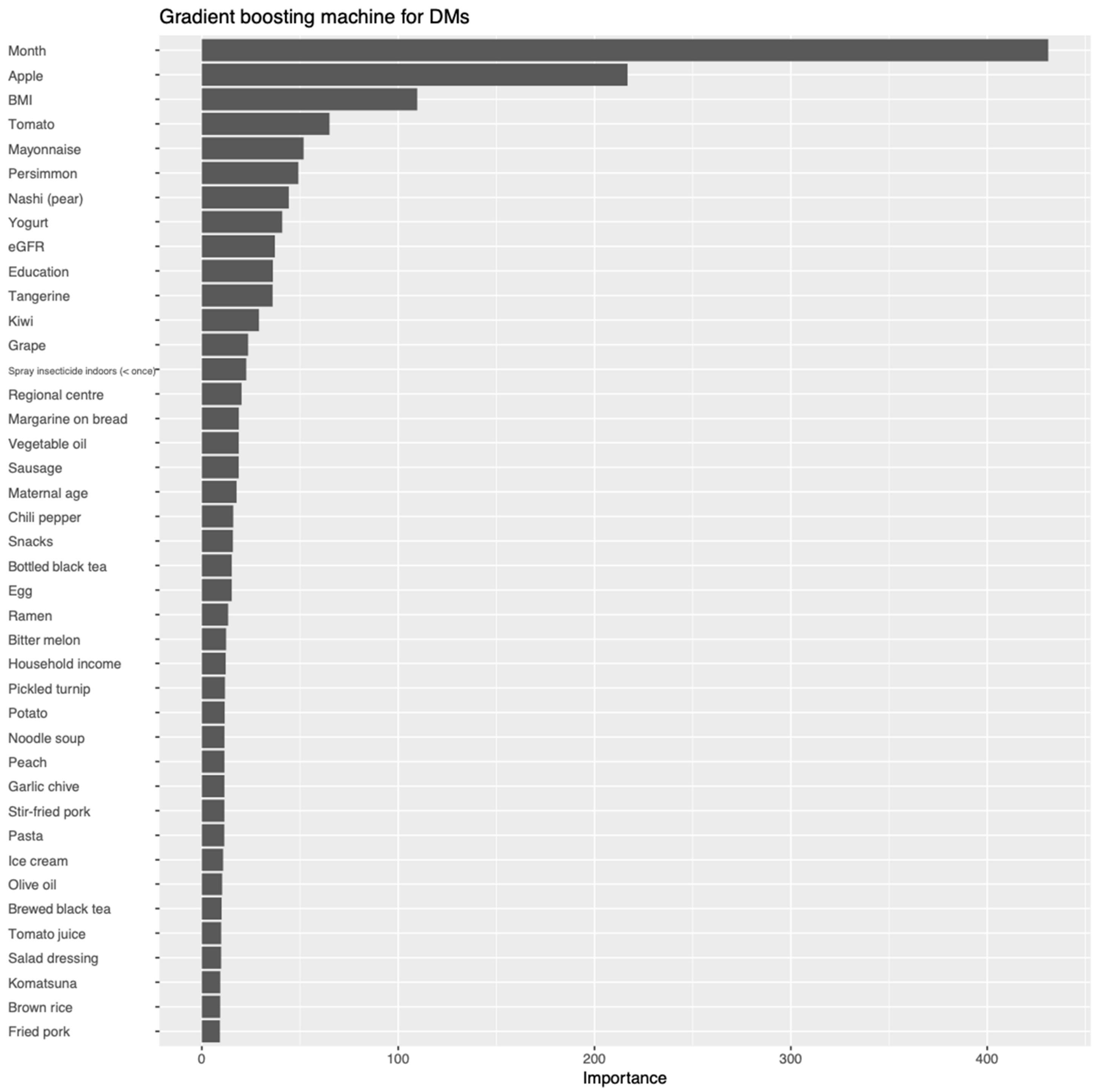

5

Figure S7. Variable importance of the important features selected by gradient boosting machine for DMs. The x-axis represents the importance value of each variable.

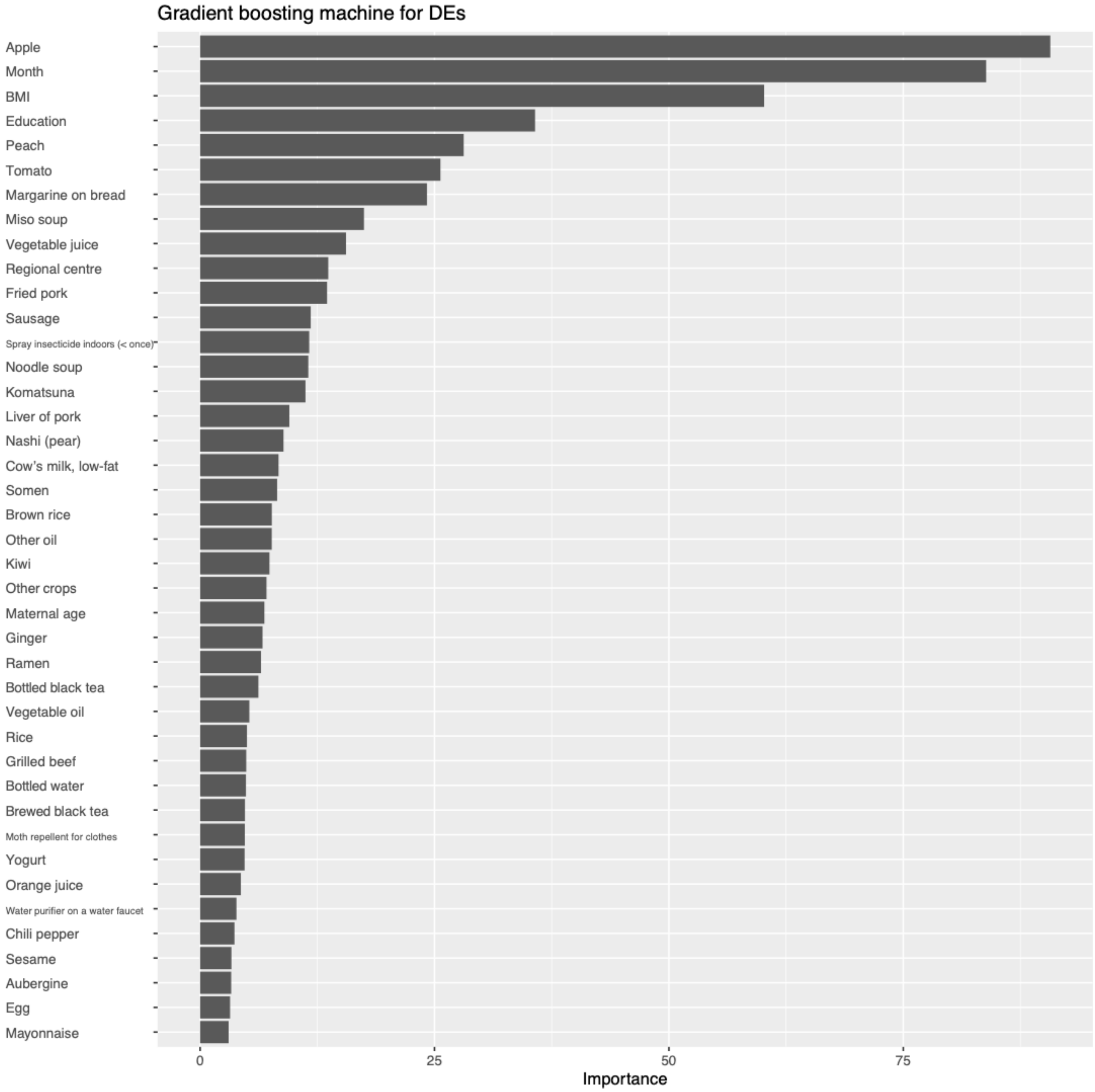

Figure S8. Variable importance of the important features selected by gradient boosting machine for DEs. The x-axis represents the importance value of each variable.
